# Brefeldin A-inhibited guanine nucleotide-exchange protein 3 (BIG3) is predicted to interact with its partner through an ARM-type α-helical structure

**DOI:** 10.1186/1756-0500-7-435

**Published:** 2014-07-06

**Authors:** Yi-An Chen, Yoichi Murakami, Shandar Ahmad, Tetsuro Yoshimaru, Toyomasa Katagiri, Kenji Mizuguchi

**Affiliations:** 1 National Institute of Biomedical Innovation, 7-6-8 Saito-asagi, Ibaraki city, Osaka 567-0085, Japan; 2 Graduate School of Information Sciences, Tohoku University, 6-3-09 Aramaki-aza-aoba, Aoba-ku, Sendai city, Miyagi 980-8579, Japan; 3 Division of Genome Medicine, Institute for Genome Research, The University of Tokushima, 3-18-15 Kuramoto-cho, Tokushima 770-8503, Japan

**Keywords:** Breast cancer, Estrogen receptor-alpha, BIG3, PHB2, Protein-protein interaction, Bioinformatics

## Abstract

**Background:**

Brefeldin A-inhibited guanine nucleotide-exchange protein 3 (BIG3) has been identified recently as a novel regulator of estrogen signalling in breast cancer cells. Despite being a potential target for new breast cancer treatment, its amino acid sequence suggests no association with any well-characterized protein family and provides little clues as to its molecular function. In this paper, we predicted the structure, function and interactions of BIG3 using a range of bioinformatic tools.

**Results:**

Homology search results showed that BIG3 had distinct features from its paralogues, BIG1 and BIG2, with a unique region between the two shared domains, Sec7 and DUF1981. Although BIG3 contains Sec7 domain, the lack of the conserved motif and the critical glutamate residue suggested no potential guaninyl-exchange factor (GEF) activity. Fold recognition tools predicted BIG3 to adopt an α-helical repeat structure similar to that of the armadillo (ARM) family. Using state-of-the-art methods, we predicted interaction sites between BIG3 and its partner PHB2.

**Conclusions:**

The combined results of the structure and interaction prediction led to a novel hypothesis that one of the predicted helices of BIG3 might play an important role in binding to PHB2 and thereby preventing its translocation to the nucleus. This hypothesis has been subsequently verified experimentally.

## Background

Breast cancer is the most common cancer among women worldwide [1]. The majorities of breast cancers are estrogen receptor-alpha (ERα) positive and depend on the hormone estrogen for growth. Estradiol (E2) is known to induce cell proliferation by binding to ERα, resulting in the transcriptional activation of its downstream genes [2,3]. Antagonists to ERα such as tamoxifen can block the effects of E2 on breast cancer cells and thereby interfere with estrogen-induced cell proliferation. Although tamoxifen has been a great success and improves breast cancer survival rates considerably [4-6], a significant proportion of ERα-positive breast cancer is tamoxifen-unresponsive, and tamoxifen-resistant cases have been also reported [7,8]. The mechanism of E2/ERα signalling is not fully understood and a better understanding of the E2/ERα pathway will be essential for more effective and alternate treatments for breast cancer.

Recently, genome-wide profiling of gene expression in breast cancer cells has identified a novel regulator of E2/ERα signalling, brefeldin A-inhibited guanine nucleotide-exchange protein 3 (BIG3). BIG3 has been shown to be over-expressed in breast cancer cells but hardly detectable in normal human tissues [8]. Small-interfering RNA (siRNA)-mediated knockdown of BIG3 was shown to suppress the growth of breast cancer cells significantly [9]. Co-immunoprecipitation and immuno-blotting assays have shown that BIG3 interacts with prohibitin 2 (PHB2), a protein that can repress the activity of ER. PHB2 was shown to be localized mainly in the cytoplasm [10]. When BIG3 is absent, E2 stimulation causes the translocation of PHB2 to the nucleus and results in the suppression of the ERα transcriptional activity. On the other hand, when BIG3 is over-expressed, PHB2 remains in the cytoplasm even with estrogen treatment and it has been shown that the intracellular localization of PHB2 is dependent on its interaction with BIG3 in the cytoplasm. Therefore, the current hypothesis is that BIG3 interacts with PHB2 and traps it in the cytoplasm and thereby prevents its nuclear translocation, resulting in increases in the transcriptional activities of ERα.

This novel mechanism of ERα regulation by BIG3 has the potential to offer molecular details of signalling events in ERα-positive breast cancer cells and can lead to new ways of therapeutic intervention. The progress has been hampered, however, by the lack of information about molecular functions of BIG3. The BIG3 protein consists of 2177 amino acid residues and its sequence suggests no association with any well-characterized protein family and provides little clues as to its molecular function. Although a series of co-immunoprecipitation assays identified residues 86-434 to be responsible for the binding of BIG3 to PHB2, further attempts at narrowing down the binding region or any other biochemical characterization had been unsuccessful until computational predictions, described in this paper, were made and subsequently verified experimentally [10].

In this paper, we describe details of our predictions for the structure, function and interactions of BIG3 using state-of-the-art bioinformatic tools. The prediction of protein interaction sites, supported by consistent fold recognition results, led to a specific hypothesis about the nature of the molecular interactions between BIG3 and PHB2, which was a key to the successful experimental verification studies.

## Results and discussion

### BIG3 has features distinct from BIG1 and BIG2

**Figure 1:**
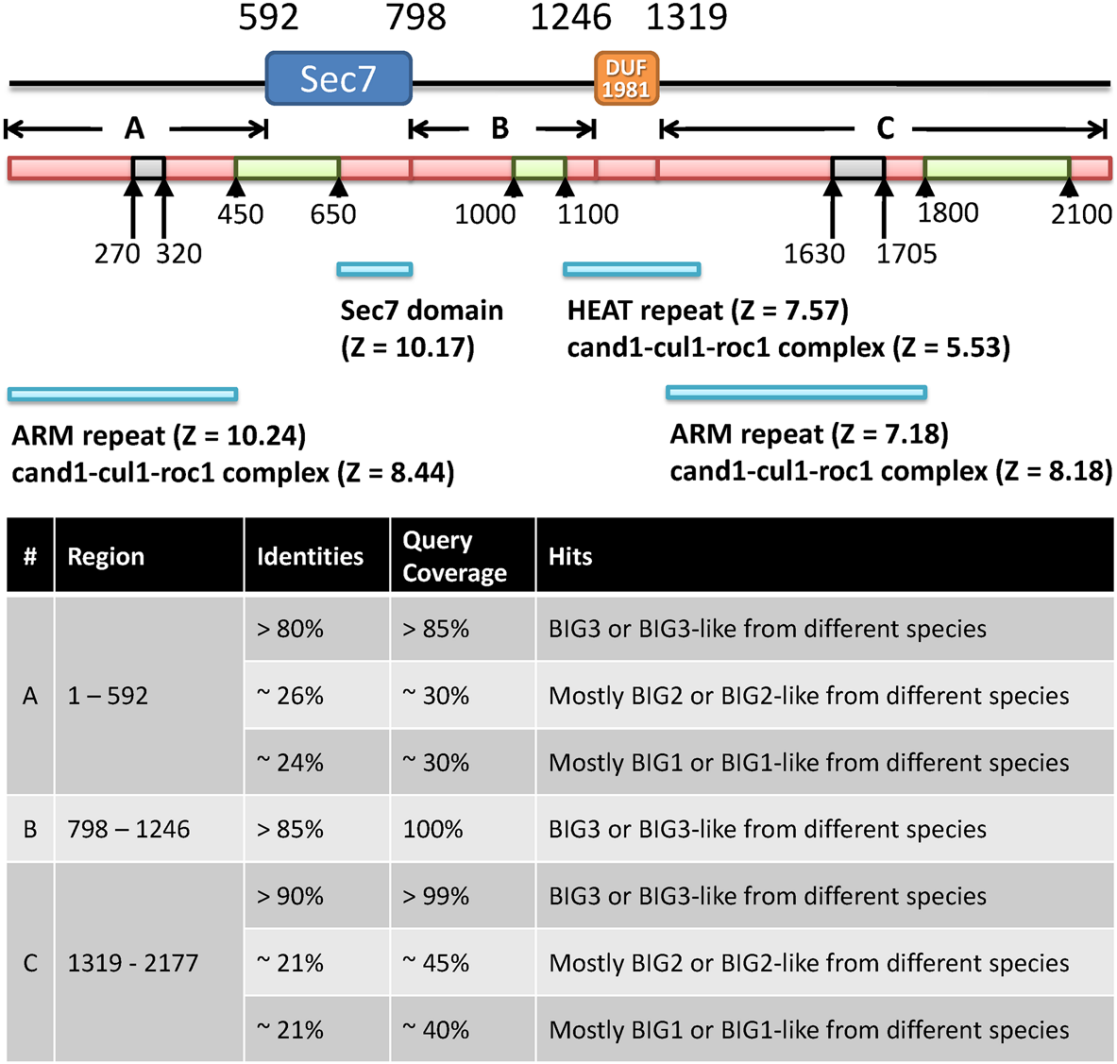
**Sequence analysis of BIG3.** The locations of the two domains identified by Pfam and SMART are shown at the top. Shown below are the results of secondary structure and disorder predictions: red, α-helical; grey, coil; and green, disorder. The three segments underneath, **A**, **B** and **C**, indicate the query sequences for the BLAST, FUGUE and HHpred searches. The FUGUE hits are shown below and the BLAST results are summarized in the table at the bottom.

The BIG3 protein consists of 2177 amino acids but standard sequence-based tools such as Pfam [11] and SMART [12] identified only two domains, Sec7 (at 592-798) and DUF1981 (at 1246-1303) (Figure 1). The Sec7 domain has been shown to be linked with guanine nucleotide exchange factor (GEF) activity [13,14], although its relevance to the biological function of BIG3 is unclear (see below). DUF1981 is a functionally uncharacterized domain defined in Pfam and it is mostly found in GEF related proteins.To gain further insights into the structure and function of BIG3, we split its amino acid sequence into three segments based on the Pfam and SMART domain assignments and ran BLAST for each segment against the non-redundant protein (nr) database. The segment before the Sec7 domain and that after the DUF1981 domain were found to be conserved both in BIG3 orthologues (annotated as BIG3 in the database) and paralogues (annotated as BIG1 and BIG2). On the other hand, the segment between the Sec7 and DUF1981 domains produced significant hits only to the orthologues (Figure 1). When the sequences of BIG3 and the human BIG family proteins were compared, BIG1 and BIG2 showed 74% identity overall and higher identities in both the Sec7 and DUF1981 domains (90%). However, BIG3 showed only 21% identities to BIG1 and BIG2, with ~30% identity in DUF1981 and no significant similarity (i.e., a BLAST e-value of > 10) found in the Sec7 domain. These observations suggested that BIG3 was the most distinct among its paralogues, with a markedly unique region between the Sec7 and DUF1981 domains.

**Figure 2:**
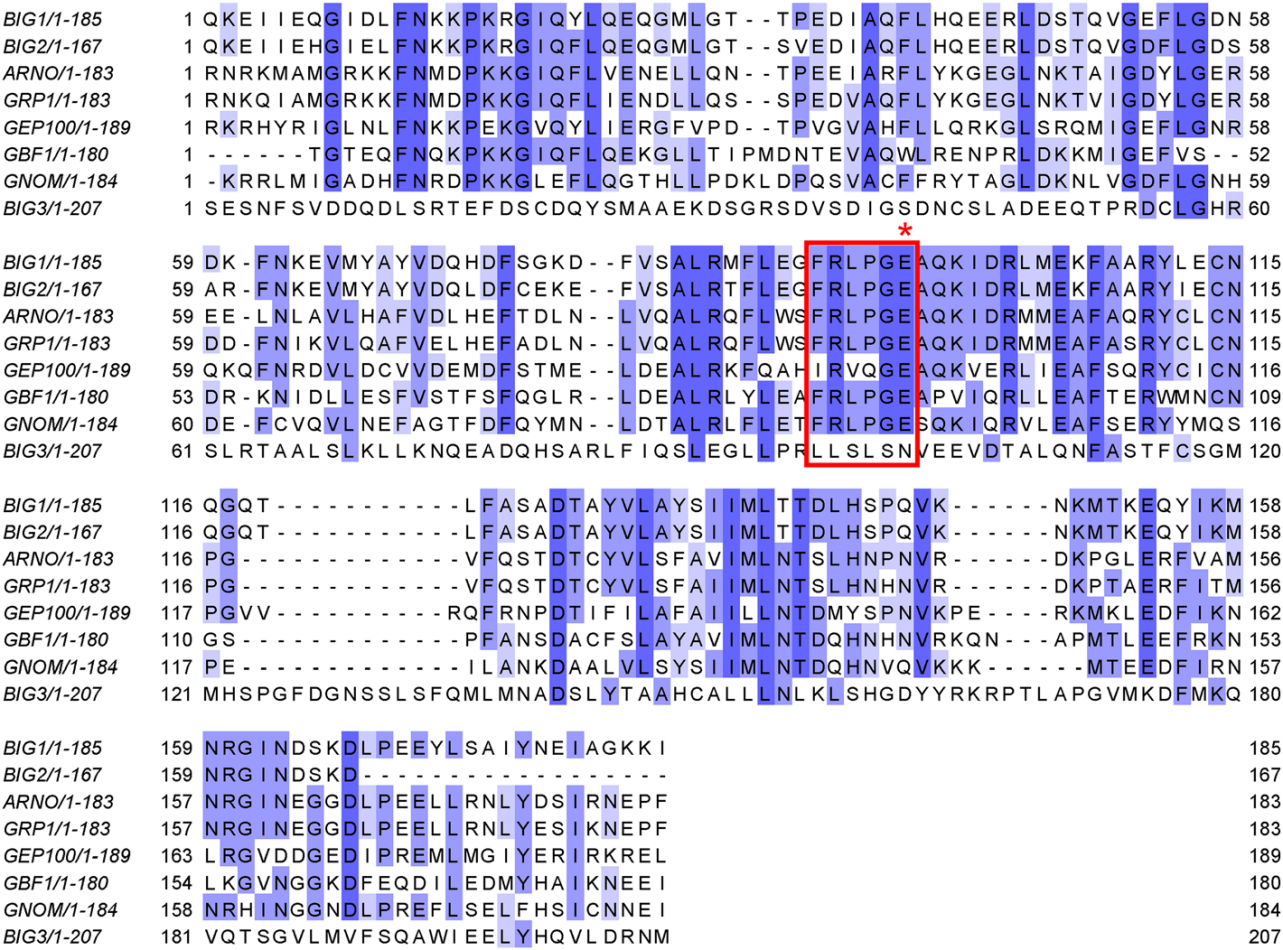
**Multiple sequence alignment of eight Sec7 domains.** Selected members of the Sec7 family were aligned by using CLUSTALW. (See Methods for the protein names and accessions.) Sequences are coloured by percentage identities: dark purple, >80%; purple, 60% ~ 80%; light purple 40% ~ 60%; white, <40%. The red box shows the conserved motif in Sec7 domain. BIG3 lacks the conserved motif, especially the critical glutamate residue (indicated by the red asterisk).

BIG3, unlike BIG1 and BIG2, potentially lacks GEF activity, despite being annotated to contain the Sec7 domain (based on sequence similarity). Sec7, first discovered in the SEC7 gene product of *S. cerevisiae*, has a central GEF domain for the ADP-ribosylation factor family involved in vesicular transport processes [15-17]. There is a highly conserved motif, FRLPGE, among the Sec7 proteins, with the last glutamate residue essential for GEF activity [18-24]. It has also been shown that the Sec7 domain alone is sufficient for GEF activity [17]. Alignments between BIG3 and known Sec7 proteins (generated with both the sequence-only method CLUSTALW [25] and the structure-based method FUGUE [26]) were stable and unambiguous in a region around the conserved motif, and they showed that BIG3 lacked the conserved motif and critically, the essential glutamate residue (Figure 2). This region is conserved among the BIG3 orthologues, all of which lack the functional motif. This observation suggests that BIG3 is unlikely to be a GEF protein, consistent with a previous demonstration by the GST-GAT pull-down assay [27].

### BIG3 is likely to adopt α-helical repeat structures similar to that of the armadillo (ARM) family

**Figure 3:**
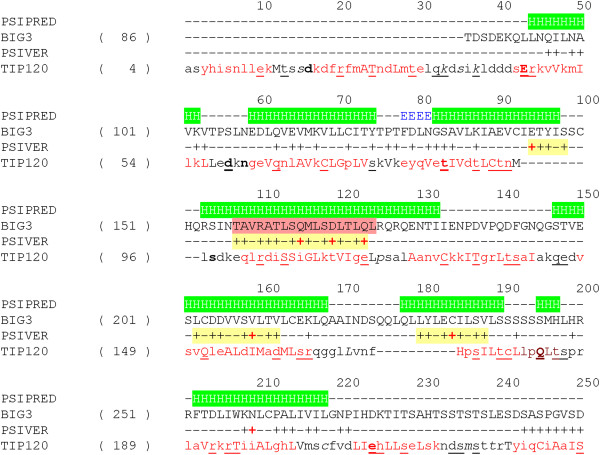
**Predicted structure and interaction sites of BIG3.** Results of secondary structure prediction (PSIPRED) and interaction site prediction (PSIVER) were mapped on a structure-based alignment (generated with FUGUE and formatted with JOY [31]) between BIG3 (residues 86-300) and TIP120 (PDB 1u6g, chain C). For the PSIPRED prediction, H stands for α-helices, E for β strands and dash for coil structures. For the PSIVER results, scores greater than 0.39 are labelled with plus (+) signs. Clusters of the highest scoring residues are highlighted with a yellow background and those residues with a score greater than 0.6 are indicated with red plus signs. The helix used for the helical wheel projection (see Figure 4) is indicated with a red background. Structural environments are annotated with JOY, the formatting convention of which is as follows: red, α-helices; blue, β strands; maroon, 3_10_ helices; upper case letters, solvent inaccessible; lower case letters, solvent accessible; bold type, hydrogen bonds to mainchain amides; underlining, hydrogen bonds to mainchain carbonyls; italic, positive mainchain torsion angles φ.

We then attempted to predict the structure of BIG3 using more specialized tools. Both POODLE [28,29] and PrDOS [30] predicted BIG3 to adopt largely well-defined three-dimensional structures, except for three intrinsically disordered regions at 450-650, 1000-1100 and 1800-2100. Secondary structure prediction by PSI-PRED suggested that BIG3 predominately consisted of helical structures (Figure 1). No evidence was obtained for any of the predicted helices to be coiled-coil or transmembrane structures. Although no repeats were detected by sequence-based methods (see Methods), fold recognition using FUGUE and HHpred suggested that some parts of BIG3 would consist of ARM (and related) repeats, with statistically significant sores (FUGUE Z-score > 7, 99% confidence and HHpred probability > 80%; see Figure 1).Despite the high confidence scores, however, generating alignments proved to be tricky; because of the nature of the α-helical repeats, slightly different alignments were produced for different hits. Figure 3 shows an alignment with the TIP120 protein, the highest scoring hit by FUGUE when queried with residues 86-434 of BIG3. TIP120 is a member of the HEAT repeat family. The HEAT repeat is related to the ARM repeat and sometimes classified as a subgroup of the ARM family. In this alignment, the predicted α-helices generally agree well with the helical positions in the TIP120 structure.

The ARM repeat, first discovered in armadillo gene of Drosophila, is an approximately 40 amino acid long tandem repeat, forming a super-helix of helices. Proteins in the ARM family are known to function in various processes, including cytoskeletal regulation, signalling, tumor suppression and nuclear translocation. It has been proposed that ARM may mediate protein-protein interactions but currently, no typical feature of target proteins is known. Of particular note is that the nuclear transport protein importin, known to recognize nuclear localization signals (NLSs), is a member of the ARM family. Given its predicted structure, BIG3 might also bind to its partners in a similar manner (see below).

### Prediction of protein binding sites suggested how BIG3 could possibly inhibit the nuclear translocation of PHB2

To pursue this possibility further, we attempted to predict protein-binding sites on BIG3 using PSIVER [32] and examined the results within the predicted ARM repeats, as these repeats fell within residues 1-250, a region that had been shown experimentally to be responsible for the binding of BIG3 to PHB2 [10].

**Figure 4:**
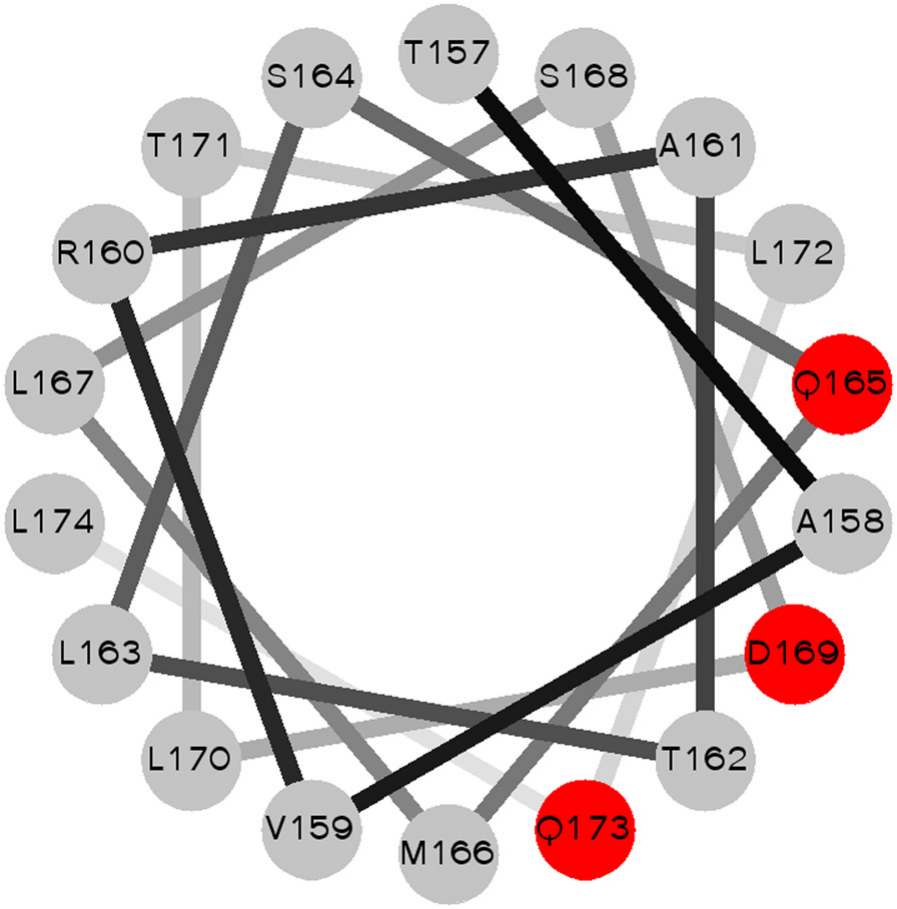
**A helical wheel projection of residues 157-174 of BIG3.** The residues with the highest predicted interaction scores, Q166, D170 and Q174 (red filled circles), are located on the same face of the helix.

Figure 3 shows the possible interaction sites on BIG3 predicted by PISVER, combined with the results of FUGUE and PSI-PRED analyses (see Additional file 1 for the raw data). The benchmark results of PSIVER showed that high scoring residues would tend to cluster together near the true interaction sites [32]. Therefore, we highlighted clusters of the highest scoring residues in Figure 3 (yellow background) and considered the residues 157-174 as the most likely interaction site (red background). This region coincides with a predicted α-helix [10], and a helical wheel projection [33] of the residues (Figure 4) shows that the side chains of the residues with locally maximal scores (red plus signs in Figure 3) sit on the same face of the helix. These results have opened a new direction for experimental research, including the construction of BIG3 mutants and the design of an inhibitory peptide. Site-directed mutagenesis showed that substituting Q165, D169 and Q173 (indicated with red plus signs in Figure 3) with alanine reduced the binding affinity to PHB2 dramatically (see Additional file 2: Figure S1 of [10]). The designed peptide, including these PHB2-binding residues, has been shown to inhibit the growth of ERα-positive breast cancer cells both *in vitro* and *in vivo*[10].

**Figure 5:**
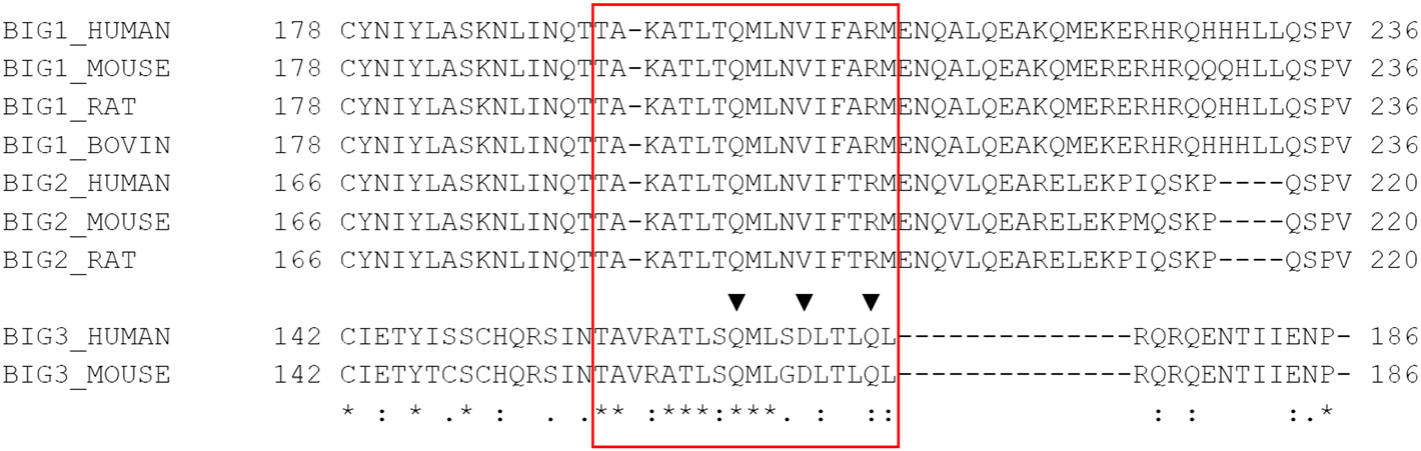
**Multiple sequence alignment of BIG3 and its homologues near the putative PHB2-binding site.** Multiple sequence alignment of the N-terminal portions (region A in Figure 1) of BIG3 and its homologues was generated by MAFFT (see Additional file 2 for the full alignment). The red box corresponds to the residues in the red background in Figure 3. The verified PHB2-binding residues are indicated with black triangles.

Since BIG1 and BIG2, the paralogues of BIG3, share some sequence similarity with BIG3 in their N-terminal portions (region A in Figure 1), we generated a multiple sequence alignment and examined the putative PHB2-binding site, including the three verified binding residues (Figure 5, red box). Because of the general sequence similarity, the alignment in this region was unambiguous and showed that, of the three PHB2-binding residues, only Q165 was conserved among BIG1, BIG2 and BIG3. Since the other two residues have been shown to be critical for PHB2 binding [10], we conclude that BIG1 and BIG2 are unlikely to form a heterodimer with PHB2, although these paralogues may still share some other common functions.

**Figure 6:**
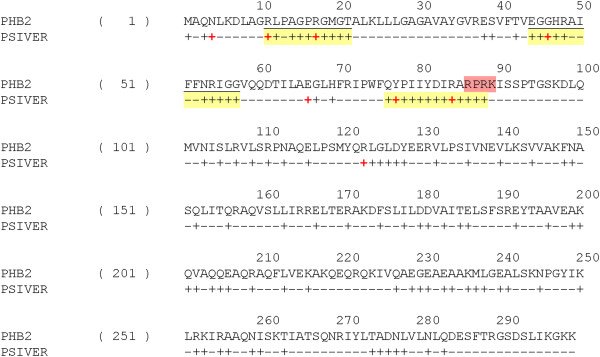
**Predicted interaction sites of PHB2.** Results of the binding site prediction for PHB2 by PSIVER. Scores greater than 0.39 are labelled with plus (+) signs. Clusters of the highest scoring residues are highlighted with a yellow background and the scores greater than 0.6 are shown in red. The NLS region, residues 86-89, is indicated with a red background. The consensus prediction results for the BIG3 binding site by PSIVER and PPiPP are underlined.

We also predicted protein-binding sites on PHB2. PSIVER predicted a few regions to be possible interacting sites (highlight in Figure 6), one of which (76-88) is close to the predicted NLS. We also used PPiPP, a recently developed neural network-based method for predicting contacting residue pairs given a pair of amino acid sequences [34]. A PPiPP search for interacting pairs between residues 1-300 of BIG3 and PHB2 (full length, 299 amino acid residues) identified R11, R17, M19, Y34, R54, I55, R88, M101 and R289 as the most likely interacting partners of the putative interacting region on BIG3 (157-173, Figure 3, yellow background). By combining this analysis and the prediction results by PSIVER, we found regions 11-21 and 44-57 to be the most likely BIG3-binding site (Figure 6, underlined). PHB2 is known to interact with several other proteins (such as COPG and PTMA), as reported in BIOGRID [35] and PPIview [36]. Whether the predicted region is indeed involved in BIG3-binding is yet to be verified experimentally.

PHB2 is known to be involved in several biological processes and found in different cellular compartments, including the nucleus, mitochondria and cell membrane [37-40]. Although the mechanism of translocation of PHB2 is still unclear, one possibility is that it is mediated by importin (or importin-like proteins), and BIG3 could possibly dislocate importin and interact with PHB2, preventing it from being transported to the nucleus. In light of this hypothesis, it is highly suggestive that BIG3 is predicted to adopt the same fold as that of importin.

## Conclusions

Based on the differences in sequence and the lack of conserved motif in the Sec7 domain, BIG3 was shown to have distinct features from its paralogues BIG1 and BIG2. Structural analysis showed that BIG3 would adopt α-helical repeat structures similar to that of the ARM family. Prediction of interaction sites between BIG3 and PHB2 provided a new insight into how BIG3 would interfere the translocation of PHB2 and suggested a specific, testable hypothesis.

## Methods

### Sequence analysis

Protein sequences of BIG3 [Swiss-Prot:Q5TH69] and PHB2 [Swiss-Prot:Q99623] were retrieved from Uniprot. Pfam (http://pfam.xfam.org/) and SMART (http://smart.embl-heidelberg.de/) searches were performed using their web servers. BLAST was run on the NCBI website (http://www.ncbi.nlm.nih.gov/BLAST/) using default parameters. A multiple sequence alignment of the Sec7 domains of BIG1 [Swiss-Prot:Q9Y6D6], BIG2 [Swiss-Prot:Q9Y6D5], ARNO [Swiss-Prot:Q99418], GBF1 [Swiss-Prot:Q92538], GRP1 [Swiss-Prot:O43739], GNOM [Swiss-Prot:Q42510], GEP100 [Swiss-Prot:Q6ND90] and BIG3 was generated using CLUSTALW and formatted by Jalview [41]. A multiple sequence alignment of the N-terminal portions of BIG3 and its homologues was generated by MAFFT version 7 (http://mafft.cbrc.jp/alignment/server/). The sequences included were human BIG1 [Swiss-Prot:Q9Y6D6] and BIG2 [Swiss-Prot:Q9Y6D5], mouse BIG1 [Swiss-Prot:G3X9K3], BIG2 [Swiss-Prot:A2A5R2] and BIG3 [Swiss-Prot:Q3UGY8], rat BIG1 [Swiss-Prot:D4A631] and BIG2 [Swiss-Prot:Q7TSU1] and bovine BIG1 [Swiss-Prot:O46382].

### Structure prediction

Secondary structure was predicted by using a local installation of PSIPRED [42] with the default script. Disordered regions were predicted using both POODLE-L and POODLE-W on the POODLE server (http://mbs.cbrc.jp/poodle/index.html) and PrDOS (http://prdos.hgc.jp/index.html). Coiled-coil was predicted using Paircoil2 (http://groups.csail.mit.edu/cb/paircoil2/) [43] and COILS (http://www.ch.embnet.org/software/COILS_form.html) [44]. Sequence repeats were predicted using REP (http://www.bork.embl.de/~andrade/papers/rep/search.html) [45], HHrep (http://toolkit.tuebingen.mpg.de/hhrep) [46] and REPRO (http://www.ibi.vu.nl/programs/reprowww/) [47]. Fold recognition was performed using FUGUE (http://tardis.nibio.go.jp/fugue/) and HHpred (http://toolkit.tuebingen.mpg.de/hhpred/, with the HMM database of pdb70_18Dec10) [48] using the three segments defined in Figure 1 as queries.

### Interaction site prediction

Interaction sites on BIG3 and PHB2 were predicted using PSIVER [32]. The default threshold of 0.390 was used in this study. Interacting pair positions between the two proteins were predicted using PPiPP [34] with default parameters.

### Helical wheel projection

The helical wheel projection was generated by a custom script derived from the original code by Zidovetzki and Armstrong [33].

## Competing interests

The authors declare that they have no competing interests.

## Authors’ contributions

KM conceived and coordinated the study. YAC carried out and summarized the computational analysis. YM and SA participated in the protein-protein interaction prediction. TY and TK participated in the discussion of biological relevance and experimental validation. YAC, YM and KM wrote the manuscript. All authors read and approved the final manuscript.

## Supplementary Material

Additional file 1 **Detailed prediction results by PSIVER for protein-binding sites on BIG3.** The columns represent: record type (always “PRED”), residue position, binary prediction (plus for the raw score above the threshold of 0.39 and minus otherwise), one-letter amino acid code, raw score and z-score, respectively. Click here for file

Additional file 2 **Multiple sequence alignment of the N-terminal portions of BIG3 and its homologues.** Multiple sequence alignment of the N-terminal portions (region A in Figure 1) of BIG3 and its homologues by MAFFT. Click here for file
