# Supplementary material for: Brefeldin A-inhibited guanine nucleotide-exchange protein 3 (BIG3) is predicted to interact with its partner through an ARM-type α-helical structure
Source: BMC Res Notes. 2014 Jul 6;7:435. doi: 10.1186/1756-0500-7-435 (PMC4096751; doi:10.1186/1756-0500-7-435)
Supplement: Additional file 2 — Multiple sequence alignment of the N-terminal portions of BIG3 and its homologues. Multiple sequence alignment of the N-terminal portions (region A in Figure 1) of BIG3 and its homologues by MAFFT. [file 1756-0500-7-435-S2.pdf]

CLUSTAL format alignment by MAFFT (v7.143b)

```
BIG1_HUMAN    1 MYEGKKTKNMFLTRALEKIL--ADKEVKKAHHSQLRKACEVALEEIKA-ETEKQSPPHGE  57
BIG1_MOUSE    1 MYEGKKTKNMFLTRALEKIL--ADKEVKKAHHSQLRKACEVALEEIKV-ETEKQSPPHGE  57
BIG1_RAT      1 MYEGKKTKNMFLTRALEKIL--ADKEVKKAHHSQLRKACEVALEEIKV-ETEKQSPPHGE  57
BIG1_BOVIN    1 MYEGKKTKNMFLTRALEKIL--ADKEVKKAHHSQLRKACEVALEEIKA-ETEKQSPPHGE  57
BIG2_HUMAN    1 -MQESQTKSMFVSRALEKIL--ADKEVKRPQHSQLRRACQVALDEIKA-EIEKQ------  50
BIG2_MOUSE    1 -MQESQTKSMFVSRALEKIL--ADKEVKRPQHSQLRRACQVALDEIKA-ELEKQ------  50
BIG2_RAT      1 -MQESQTKSMFVSRALEKIL--ADKEVKRPQHSQLRRACQVALDEIKA-ELEKQ------  50
BIG3_HUMAN    1 ---------------MEEILRKLQKEASGSKYKAIKESCTWALETLGGLDTIVKIPPH--  43
BIG3_MOUSE    1 ---------------MEEILRKLQRDASGSKYKAIKESCTWALETLGGLDTVVKIPPH--  43
                               :*:**   :::.. .::. ::.:*  **: :   :   :

BIG1_HUMAN   58 AKAGSSTLPPVKSKTNFIEADKYFLPFELACQSKCPRIVSTSLDCLQKLIAYGHLTGNAP 117
BIG1_MOUSE   58 AKAGSGTLPPVKSKTNFIEADKYFLPFELACQSKCPRIVSTSLDCLQKLIAYGHLTGRAP 117
BIG1_RAT     58 AKAGSGTLPPVKSKTNFIEADKYFLPFELACQSKCPRIVSTSLDCLQKLIAYGHLTGSAP 117
BIG1_BOVIN   58 AKAGSSTLPPVKSKTNFIEADKYFLPFELACQSKCPRIVSTSLDCLQKLIAYGHLTGNAP 117
BIG2_HUMAN   51 -RLGTAAPP----KANFIEADKYFLPFELACQSKSPRVVSTSLDCLQKLIAYGHITGNAP 105
BIG2_MOUSE   51 -RLGAAAPP----KANFIEADKYFLPFELACQSKSPRVVSTSLDCLQKLIAYGHITGNAP 105
BIG2_RAT     51 -RLGAAAPP----KANFIEADKYFLPFELACQSKSPRVVSTSLDCLQKLIAYGHITGNAP 105
BIG3_HUMAN   44 -----------------VLREKCLLPLQLALESKNVKLAQHALAGMQKLLSEERFVSMET  86
BIG3_MOUSE   44 -----------------LLREKCLLPLQLALESKNVKLAQHALAGMQKLLSEERFVSMET  86
                                 :  :* :**::** :**  ::.. :*  :***::  ::..  .

BIG1_HUMAN  118 DSTTPGKKLIDRIIETICGCFQGPQTDEGVQLQIIKALLTAVTSQHIEIHEGTVLQAVRT 177
BIG1_MOUSE  118 DSTTPGKKLIDRIIETICGCFQGPQTDEGVQLQIIKALLTAVTSQHIEIHEGTVLQAVRT 177
BIG1_RAT    118 DSTTPGKKLIDRIIETICGCFQGPQTDEGVQLQIIKALLTAVTSQHIEIHEGTVLQAVRT 177
BIG1_BOVIN  118 DSTTPGKKLIDRIIETICGCFQGPQTDEGVQLQIIKALLTAVTSQHIEIHEGTVLQAVRT 177
BIG2_HUMAN  106 DSGAPGKRLIDRIVETICSCFQGPQTDEGVQLQIIKALLTAVTSPHIEIHEGTILQTVRT 165
BIG2_MOUSE  106 DSGAPGKRLIDRIVETICNCFQGPQTDEGVQLQIIKALLTAVTSPHIEIHEGTILQTVRT 165
BIG2_RAT    106 DSGAPGKRLIDRIVETVCNCFQGPQTDEGVQLQIIKALLTAVTSPHIEIHEGTILQTVRT 165
BIG3_HUMAN   87 DSDE--KQLLNQILNAV---KVTPSLNEDLQVEVMKVLLCITYTPTFDLNGSAVLKIAEV 141
BIG3_MOUSE   87 DSDE--KQLLNQILNAV---KVTPSLNEDLQVEVMKVLLCITYTPTFDMNGSAVLKIAEV 141
                **    *:*:::*::::      *. :*.:*:::*.**   . :  :::: .::*: ...

BIG1_HUMAN  178 CYNIYLASKNLINQTTA-KATLTQMLNVIFARMENQALQEAKQMEKERHRQHHHLLQSPV 236
BIG1_MOUSE  178 CYNIYLASKNLINQTTA-KATLTQMLNVIFARMENQALQEAKQMERERHRQQQHLLQSPV 236
BIG1_RAT    178 CYNIYLASKNLINQTTA-KATLTQMLNVIFARMENQALQEAKQMERERHRQQHHLLQSPV 236
BIG1_BOVIN  178 CYNIYLASKNLINQTTA-KATLTQMLNVIFARMENQALQEAKQMEKERHRQHHHLLQSPV 236
BIG2_HUMAN  166 CYNIYLASKNLINQTTA-KATLTQMLNVIFTRMENQVLQEARELEKPIQSKP----QSPV 220
BIG2_MOUSE  166 CYNIYLASKNLINQTTA-KATLTQMLNVIFTRMENQVLQEARELEKPMQSKP----QSPV 220
BIG2_RAT    166 CYNIYLASKNLINQTTA-KATLTQMLNVIFTRMENQVLQEARELEKPIQSKP----QSPV 220
BIG3_HUMAN  142 CIETYISSCHQRSINTAVRATLSQMLSDLTLQL--------------RQRQENTIIENP- 186
BIG3_MOUSE  142 CIETYTCSCHQRSINTAVRATLSQMLGDLTLQL--------------RQRQENTIIENP- 186
                * : * .* :  . .** :***:***. :  ::               : :     :.*

BIG1_HUMAN  237 SHHEPESPQLRYLPPQTVDHISQEHEGDLDLHTNDVDKSLQDDTEPENGSDISSAENEQT 296
BIG1_MOUSE  237 SHHEPESPHLRYLPPQTVDHINQEHEGDLEPQTHDVDKSLQDDTEPENGSDISSAENEQT 296
BIG1_RAT    237 SHHEPESPHLRYLPPQTVDHIAQEQEGDLDPQTHDVDKSLQDDIEPENGSDISSAENEQT 296
BIG1_BOVIN  237 SHHEPESPQLRYLPPQTVDHIPQEHEGDLDPQTNDVDKSLQDDTEPENGSDISSAENEQT 296
BIG2_HUMAN  221 IQAAAVSPKFVRLKHSQAQSKPTTPE----------KTDLTNGEHARSDSGKVSTENGDA 270
BIG2_MOUSE  221 IQATAGSPKFSRLKQSQAQSKPTTPE----------KAELPNGDHAQSGLGKVSLENGEA 270
BIG2_RAT    221 IQATAGSPKFSRLKQSQAQSKPTTPE----------KTELPNGDHARSSLGKVNSENGEA 270
BIG3_HUMAN  187 -----DVPQDFGNQGSTVESLCDDVVSVLTVLCEKLQAAINDSQQLQ------LLYLECI 235
BIG3_MOUSE  187 -----DAPQEFRSQGLTVEALCDDVISVLAVLCEKLQASINDSQQLQ------LLYLECI 235
                       *:        .:                 .  : :. . .
```

```
BIG1_HUMAN  297 EADQATAAETLSKNEVLYDGENHDCEEKPQDIVQNIVEEMVNIVVGDMGEGTTIN----- 351
BIG1_MOUSE  297 EADQATAAETLSKNDILYDG---DYEEKPLDIVQSIVEEMVNIIVGDMGEGMAIS----- 348
BIG1_RAT    297 EADQATAAETLSKDDVLCDG---ECEEKPQDIVQSIVEEMVDIIVGDMGEGTAVS----- 348
BIG1_BOVIN  297 EADQATAAETLSKNDILYDGENHDCEEKPQDIVQSIVEEMVNIVVGDTGERTTIN----- 351
BIG2_HUMAN  271 PRERGSSLS--------------GTDDGAQEVVKDILEDVVTSAIKEAAEKHGLTEPERV 316
BIG2_MOUSE  271 PRERGSPVSGRAEPSR-------GTDSGAQEVVKDILEDVVTSAVKEAAEKHGLPEPDRA 323
BIG2_RAT    271 HRERGSSISGRAEPSG-------GSDNGAQEVVKDILEDVVTSAVKEAAEKQGLPEPDQA 323
BIG3_HUMAN  236 LSVLSSSSSSMHLHRRFTDL-----------IWKNLCPALIVILGNPIHDKTITS----- 279
BIG3_MOUSE  236 LSVLSSSSSSMHLHRGFTDL-----------IWKSLCPALVVILGNPIHDKTITS----- 279
                    .:. .                      : :.:   ::        :

BIG1_HUMAN  352 -ASADGNIGTIEDGSDSENIQANGIPGTPISVAYTPSLPDDRLSVSSNDTQESGNSSGPS 410
BIG1_MOUSE  349 -ASTEGNTGTVEDGSDSENIQANGIPGTPISVAYTPSLPDDRLSVSSNDTQESGNSSGPS 407
BIG1_RAT    349 -ASADGNAGAVEDGSDSENVQANGIPGTPISAAYTPSLPDDRLSVSSNDTQESGNSSGPS 407
BIG1_BOVIN  352 -VSADGNNGTIEDGSDSENIQANGIPGTPISVAYTPSLPDDRLSVSSNDTQESGNSSGPS 410
BIG2_HUMAN  317 LGELECQECAIPPGVD-ENSQTNGI-------------ADDRQSLSSADNLES-DAQGHQ 361
BIG2_MOUSE  324 LGALECQECAVPPGVD-ENSQTNGI-------------ADDRQSLSSADNLEP-DVQGHQ 368
BIG2_RAT    324 PGVPECQECTVPPAVD-ENSQTNGI-------------ADDRQSLSSADNLEP-DAQGHP 368
BIG3_HUMAN  280 -AHTSSTSTSLESDSASPGVSDHGRGSGCSCTAPALSGP--------------------- 317
BIG3_MOUSE  280 -AHSTSTSTSMESDSASLGVSDHGRGSGCSCTAPTLSGP--------------------- 317
                         ::       . . :*              .

BIG1_HUMAN  411 PGAKFSHILQKDAFLVFRSLCKLSMKPLSDGPPDPKSHELRSKILSLQLLLSILQNAGPI 470
BIG1_MOUSE  408 PGAKFSHILQKDAFLVFRSLCKLSMKPLSDGPPDPKSHELRSKILSLQLLLSILQNAGPV 467
BIG1_RAT    408 PGAKFSHILQKDAFLVFRSLCKLSMKPLSDGPPDPKSHELRSKILSLQLLLSILQNAGPV 467
BIG1_BOVIN  411 PGAKFSHILQKDAFLVFRSLCKLSMKPLSDGPPDPKSHELRSKILSLQLLLSILQNAGPI 470
BIG2_HUMAN  362 VAARFSHVLQKDAFLVFRSLCKLSMKPLGEGPPDPKSHELRSKVVSLQLLLSVLQNAGPV 421
BIG2_MOUSE  369 VAARFSHILQKDAFLVFRSLCKLSMKPLGEGPPDPKSHELRSKVVSLQLLLSVLQNAGPV 428
BIG2_RAT    369 VAARFSHILQKDAFLVFRSLCKLSMKPLGEGPPDPKSHELRSKVVSLQLLLSVLQNAGPV 428
BIG3_HUMAN  318 ---------------VARTIYYIAAELV----------RLVGSVDSMKPVLQSLYHRVLL 352
BIG3_MOUSE  318 ---------------VARTIYYLAAELV----------RLVGSVDSMKPVLQSLYHRVLL 352
                               * *::  :: : :          .* ..: *:: :*. * :   :

BIG1_HUMAN  471 FRTNEMFINAIK---------QYLCVALSKNGVSSVPEVFELSLSIFLTLLSNFKTHLKM 521
BIG1_MOUSE  468 FRTNEMFINAIK---------QYLCVALSKNGVSSVPEVFELSLSIFLTLLSNFKTHLKM 518
BIG1_RAT    468 FRTNEMFINAIK---------QYLCVALSKNGVSSVPEVFELSLSIFLTLLSNFKTHLKM 518
BIG1_BOVIN  471 FGTNEMFINAIK---------QYLCVALSKNGVSSVPEVFELSLSIFLTLLSNFKTHLKM 521
BIG2_HUMAN  422 FRTHEMFINAIK---------QYLCVALSKNGVSSVPDVFELSLAIFLTLLSNFKMHLKM 472
BIG2_MOUSE  429 FRSHEMFVTAIK---------QYLCVALSKNGVSSVPDVFELSLAIFLTLLSNFKMHLKM 479
BIG2_RAT    429 FRSHEMFVTAIK---------QYLCVALSKNGVSSVPDVFELSLAIFLTLLSNFKMHLKM 479
BIG3_HUMAN  353 YPPPQHRVEAIKIMKEILGSPQRLCDLAGPSSTESESRKRS---------ISKRKSHL-- 401
BIG3_MOUSE  353 YPPPQHRVEAIKIMKEILGSPQRLYDLAGPSSIESEPRKRS---------ISKRKSHL-- 401
                : . :  : ***         * *    . .. .* .   .         :*: * **

BIG1_HUMAN  522 QIEVFFKEIFLYILETSTSSFDHKWMVIQTLTRICADAQSVVDIYVNYDCDLNAANIFER 581
BIG1_MOUSE  519 QIEVFFKEIFLYILETSTSSFDHKWMVIQTLTRICADAQSVVDIYVNYDCDLNAANIFER 578
BIG1_RAT    519 QIEVFFKEIFLYILETSTSSFDHKWMVIQTLTRICADAQSVVDIYVNYDCDLNAANIFER 578
BIG1_BOVIN  522 QIEVFFKEIFLYILETSTSSFDHKWMVIQTLTRICADAQSVVDIYVNYDCDLNAANIFER 581
BIG2_HUMAN  473 QIEVFFKEIFLNILETSTSSFEHRWMVIQTLTRICADAQCVVDIYVNYDCDLNAANIFER 532
BIG2_MOUSE  480 QIEVFFKEIFLNILETSTSSFEHRWMVIQTLTRICADAQCVVDIYVNYDCDLNAANIFER 539
BIG2_RAT    480 QIEVFFKEIFLNILETSTSSFEHRWMVIQTLTRICADAQCVVDIYVNYDCDLNAANIFER 539
BIG3_HUMAN  402 ---------------------DLLKLIMDGMTEAC--------IKGGIEACYAAVSCVCT 432
BIG3_MOUSE  402 ---------------------DLLKLIMDGMTEAC--------IKGGIEACYAAVSCVCT 432
                                     :   :::: :*. *        *  . :.   *.. .
```

```
BIG1_HUMAN  582 LVNDLSKIAQGRGSQELGMSNVQELSLR----KKGLEC---LVSILKCMVEWSKDQYVNP 634
BIG1_MOUSE  579 LVNDLSKIAQGRGSQELGMSNVQELSLR----KKGLEC---LVSILKCMVEWSKDQYVNP 631
BIG1_RAT    579 LVNDLSKIAQGRGSQELGMSNVQELSLR----KKGLEC---LVSILKCMVEWSKDQYVNP 631
BIG1_BOVIN  582 LVNDLSKIAQGRGSQELGMSNVQELSLR----KKGLEC---LVSILKCMVEWSKDQYVNP 634
BIG2_HUMAN  533 LVNDLSKIAQGRSGHELGMTPLQELSLR----KKGLEC---LVSILKCMVEWSKDLYVNP 585
BIG2_MOUSE  540 LVNDLSKIAQGRSGHELGMTPLQELSLR----KKGLEC---LVSILKCMVEWSKDLYVNP 592
BIG2_RAT    540 LVNDLSKIAQGRSGHELGMTPLQELSLR----KKGLEC---LVSILKCMVEWSKDLYVNP 592
BIG3_HUMAN  433 LLGALDELSQGKG---LSEGQVQLLLLRLEELKDGAEWSRDSMEINEADFRWQRRVLSSE 489
BIG3_MOUSE  433 LLGALDELSQGKG---LNDTQVQQLLLRLEELRDGAESSRDSMEINEADFRWQRRVLSSE 489
                *:. *.:::**:.   *.   :* * **    :.* *     :.* :. ..*.:    .

BIG1_HUMAN  635 NSQTTLGQEKPSEQEMSEIKHPETINRYGSLNSLESTSSSGIGSYSTQMSGTDNPEQFEV 694
BIG1_MOUSE  632 NSQTTLGQEKPSEQEISEVKHPETINRYGSLNSLESTSSSGIGSYSTQMSGTDNPE---- 687
BIG1_RAT    632 NSQTTLGQEKPSEQEISEIKHPETINRYGSLNSLESTSSSGIGSYSTQMSGTDNPE---- 687
BIG1_BOVIN  635 NSQTTLGQEKPSEQETSEMKHPETINRYGSLNSLESTSSSGIGSYSTQMSGTDNPEQFEV 694
BIG2_HUMAN  586 NHQTSLGQERLTDQEIGDGKGLD-MARRCSVTSMESTVSSG-----TQTTVQDDPEQFEV 639
BIG2_MOUSE  593 NHQATLGQERLPDQEMGDGKGLD-MARRCSVTSVESTVSSG-----TQTAIQDDPEQFEV 646
BIG2_RAT    593 NHQATLGQERLPDQEMGDGKGLD-MARRCSVTSVESTVSSG-----TQTAIPDDPEQFEV 646
BIG3_HUMAN  490 HTPWESGNERSLDISISVTTDTGQTTLEGELG--QTTPEDHSGNHKNSLKSPAIPEGKET 547
BIG3_MOUSE  490 HTPWESGNERSPDISISVTTDTGQTTLEGELG--QTTPED----HKNGLKSPAIQEGKGT 543
                :     *:*:  : . .  .         .:   ::* ..      .        *

BIG1_HUMAN  695 LKQ--------QKEIIEQG-------------IDL 708
BIG1_MOUSE      -----------------------------------
BIG1_RAT        -----------------------------------
BIG1_BOVIN  695 LKQ--------QKEIIEQG-------------IDL 708
BIG2_HUMAN  640 IKQ--------QKEIIEHG-------------IEL 653
BIG2_MOUSE  647 IKQ--------QKEIIEHG-------------IEL 660
BIG2_RAT    647 IKQ--------QKEIIEHG-------------IEL 660
BIG3_HUMAN  548 LSKVLETEAVDQPDVVQRSHTVPYPDITNFLSVDC 582
BIG3_MOUSE  544 MGKVSEPEAIDQPDVVQRSHTVPYPDITNFLSVDC 578
```

Method

L-INS-i (Probably most accurate, very slow)
% mafft --inputorder --auto input
